# Magnitude of elevated iron stores and risk associated in steady state sickle cell anemia Congolese children: a cross sectional study

**DOI:** 10.1186/s12878-019-0134-7

**Published:** 2019-02-08

**Authors:** Jean-Robert Makulo, Karen Efombola Itokua, Rosette Kevani Lepira, Gloire Mavinga Bundutidi, Michel Ntetani Aloni, René Makuala Ngiyulu, Jean Lambert Gini, François Bompeka Lepira

**Affiliations:** 1 Division of Nephrology, Nephrology Unit, Department of Internal Medicine, University Hospital of Kinshasa, Faculty of Medicine, University of Kinshasa, Kinshasa XI, PO.BOX 123, Democratic Republic of Congo; 2 Division of Hemato-Oncology and Nephrology, Department of Pediatric, University Hospital of Kinshasa, University of Kinshasa, Kinshasa, Democratic Republic of Congo

**Keywords:** Sickle cell anemia, Elevated iron stores, Polytransfusions, DR Congo

## Abstract

**Background:**

The serum ferritin assay is recommended in Sickle Cell Anemia (SCA) patients receiving regular transfusions. According to several authors, elevated iron stores indicating iron chelation corresponds to hyperferritinemia ≥500 ng/ml, and becomes detectable after twenty blood transfusions. The objectives of the study were to determine the prevalence of elevated iron stores and identify associated risk factors in a case series of Steady state SCA Congolese children.

**Material and methods:**

Serum ferritin was assayed in Steady state SCA children followed in 2 specialized hospitals in Kinshasa. Elevated iron stores was defined as serum ferritin level ≥ 500 ng/ml, and the associated risk factors were identified using univariate analysis.

**Results:**

Seventy patients (median age 9 years, 56% boys, 53% receiving hydroxyurea) were selected in the study. Serum ferritin levels ranged from 24 to 2584 ng / ml with 21.4% of children having elevated iron stores. Mean levels of LDH, indirect bilirubin, plasma free Hb and CRP were similar between the 2 groups whereas history of polytransfusions (> 3 during the last year) was more frequent among patients with elevated iron stores (73% vs. 44%, *p* = 0.078). Receiving > 3 transfusions in a year vs. 0 was the main risk factor associated with elevated iron stores [OR 6.17 (95% CI: 1.81–20.96)].

**Conclusion:**

In SCA children, hyperferritinemia requiring iron chelation is most strongly related to blood transfusion. This situation concerned almost one in five children in present study; this shows the magnitude of the problem which is underestimated.

## Introduction

Sickle cell anemia (SCA) is the most common genetic disease in populations of African descent [1]. According to African regions and tribes, the homozygous form affects 1 to 2% of live births, whereas the heterozygous form can affect 10 to 35% of individuals [2, 3]. The hyperhemolytic crisis, one of the most common complications of this disease, usually requires urgent transfusion therapy, which exposes to iron overload since each transfused red blood cell provides about 200 mg of iron [4].

The management of sickle cell disease guidelines (The National Heart Lung and Blood Institute) recommends monitoring and treatment of iron overload in any patient who is regularly transfused [5]. Serum ferritin is the most frequently used test to estimate iron overload [5]. In steady state, several studies show a significant correlation between total transfused units by simple top-up and serum ferritin [6]. For the World Health Organization (WHO), the risk of iron overload in patients over 5 years-of-age is elevated beyond 200 ng / ml [7]. However, organic lesions may occur at ferritin levels above 1000 ng / ml, and some authors recommend chelation as soon as ferritin levels exceed 500 ng / ml [8].

The Democratic Republic of the Congo (DRC) has the third highest population of SCA patients in the world after Nigeria and India. However, SCA is not yet really regarded as a healthcare priority, despite WHO resolution [9]. Children with SCA are known to have an increased risk of blood transfusion and iron overload, especially in areas with holoendemic malaria like the DRC. Previous studies have reported that transfusions because of severe anaemia have been necessary in children with SCA [10–13]. Despite these high prevalence, few studies focused on the assessment of iron homeostasis in steady state SCA children. The dosage of ferritin for SCA is not common practice in DRC and is only possible in a few major health institutions in the country, meaning that diagnosis of iron overload and its organ damage are underestimated and delayed. In our midst, Tshilolo et al. reported that 35% of homozygous sickle cell children had ferritin levels above 300 ng / ml [14]. However, the number of those who require iron chelation is unknown. The objectives of this study were to estimate the prevalence of elevated iron stores and to identify the associated risk factors in a population of SCA children followed in 2 hospitals in Kinshasa specialized in the management of Sickle cell disease.

## Material and methods

The present study is a post-hoc analysis that uses the results of an oxidative stress study in homozygous SCA Congolese children whose methodological procedures have been detailed in previous publications [15, 16]. Briefly, SCA children between 2 and 18 years-of-age consecutively followed in two hospitals in the country’s capital Kinshasa, namely the Centre Médical Monkole and the Saint-Crispin Medical were selected for the period from 15 June, 2014 to 30 August, 2014. These health facilities are specialized in the care management of SCA. Only Steady state children (exlusion of SCA children who were transfused, hospitalized and had a major vaso-occlusive crisis within the last 2 months before the study) had been selected. Children with a history of liver disease or chronic alcoholism were excluded.

Serum ferritin (ng/ml) was assayed by the enzyme immunoassay using a Mini vidas® device (BioMérieux, France). The blood count, total and direct bilirubin, assay of C reactive protein (CRP), lactate dehydrogenase **(**LDH) and serum iron were performed using a Cobas C111® device (Roche, Switzerland). Plasma free hemoglobin (Hb) was assayed spectrophotometrically using a Genesys 20® device (Thermo Fisher Scientific, USA). In the present study, elevated iron stores (hyperferritinemia) was defined as ≥500 ng/ml [8]. The normal rates of serum iron were defined as values between 10 and 30 μmol/l.

### Ethical approval

Ethical approval for the study was granted by the institutional review boards of the Centre Médical Monkole (006 CEFA-MONKOLE/2014) in line with the principles of the Declaration of Helsinki, second revision. The aim and study procedures were explained to the parents or legal guardians and they provided written consent before any of the subjects where included.

### Statistical analysis

Comparisons of groups were analyzed using the Chi Square test or Fisher Exact test for the categorical variables, and the Student or Mann Whitney tests for the numerical variables according to whether the distribution of the variables was Gaussian or not Gaussian. The risk factors associated with elevated iron stores were investigated in univariate analysis and considered significant at the 5% level of significance (*p* < 0.05). We performed logistic regression analysis which evaluated the following variables: lack of hydrea, inflammatory satus (CRP > 6 mg/l and CRP > 12 mg/l), hemolytic status (LDH > median value and plasma free Hb > median value), age > 5 years and receiving transfusions vs 0 transfusion in a year.

## Results

A total of 70 SCA children (56% boys, 53% receiving hydroxyurea = HU) were selected. Their median age was 9 years with an interquartile range (IQR) of 6 to 13 years. In the past 12 months, half of children (50%) had received more than 3 transfusions, 37% had received 1–3 transfusions, and 13% had not been transfused (Table 1).

**Table 1:** Clinical and biological profile of the study population

	Whole group	male *n* = 39	female *n* = 31	*p*
Age, years	9(6–13.3)	10(7–14)	9(5–12)	0.789
Transfusions > 3/year	35(50)	23(59)	12(39)	0.189
1–3/year	26(37)	13(33)	13(42)	
0/year	9(13)	3(8)	6(19)	
WBC/mm^3^	11.618 ± 4.621	11.339 ± 4.114	12.011 ± 5.311	0.568
Platelets × 10^3^ /mm^3^	380.727 ± 154.522	364.359 ± 132.846	401.884 ± 184.553	0.344
Reticulocytes, %	12.0 (8.5–17.6)	11.0 (8.5–18.0)	13.0 (8.0–17.8)	0.799
CRP, mg/l	3.43 (2.01–5.07)	3.44 (2.52–5.99)	3.36 (1.88–5.01)	0.891
Total Bilirubin, mg/dl Indirect Bilirubin, mg/dl	1.9 (1.5–3.5) 1.9 (0.9–3.5)	2.5 (1.6–4.2) 1.9 (1.0–3.6)	1.9 (1.5–3.5) 1.3 (0.9–3.2)	0.841 0.823
Direct Bilirubin, mg/dl	0.5 (0.4–0.7)	0.6 (0.4–0.7)	0.5 (0.4–0.7)	0.680
LDH, UI/l	544(400–771)	550(424–795)	516(375–686)	1.000
Serum iron, μmol/l	16.2 (13.2–19.2)	16.2 (13.6–19.0)	16.3 (11.7–20.3)	0.941
Serum ferritin, ng/ml	209(106–453)	184(97–440)	213(106–519)	0.891
Free plasma Hb, mg/l	168(116–267)	168(110–282)	160(120–244)	0.950

Record values are expressed as absolute frequency (%), mean ± standard deviation or median (IQR 25–75)

Abbreviations: *WBC* white blood cells, *CRP* C reactive protein, *LDH* Lactate dehydrogenase, *Hb* hemoglobin

Ferritin levels ranged from 24 to 2584 ng / ml. Serum ferritin levels ≥500 ng / ml and ≥ 1000 ng / ml were found in 15 children (21.4%) and 4 children (5.7%), respectively. Among children with serum ferritin levels ≥500 ng / ml, 93.3% had normal iron level while 29 and 14% of them had CRP level ≥ 6 mg / l and ≥ 12 mg / l, respectively. Polytransfusion (> 3 in the last 12 months) was associated with hyperferritinemia ≥500 ng/l; high CRP levels appeared to be associated with hyperferritinemia, but the difference was not statistically significant (Table 2).

**Table 2:** Serum Ferritin level-based characteristics of SCA children

	Serum ferritin		*p*
	< 500 ng/ml (*n* = 55)	≥ 500 ng/ml (*n* = 15)	
male, %	31(57)	8(53)	0.976*
Age < 5 years, %	10(18)	5(33)	0.286*
HU, %	26(48)	10(67)	0.247*
Major VOC ≥ 2/an, %	6(11)	3(20)	0.391*
Transfusions > 3/year, %	24(44)	11(73)	0.078*
Hct, %	22.4 (20.7–24.8)	22.4 (19.0–28.1)	0.903
Platelets ×10^3^ /mm^3^	387 (293–472)	288 (172–427)	0.239
WBC, ×10^3^ élts/mm^3^	11.5 (8.4–14.6)	9.9 (7.2–17.6)	0.951
Reticulocytes, %	11.9 (8.0–17.0)	13.0 (9.5–23.3)	0.964
CRP, mg/l	3.4 (2.0–4.8)	3.8 (2.3–8.5)	0.764
CRP ≥ 6 mg/l, %	7(13)	4(29)	0.233*
CRP ≥ 12 mg/l, %	3(5)	2(13)	0.290*
Indirect Bilirubin, mg/dl	1.9 (1.0–3.6)	1.3 (0.9–2.0)	0.547
LDH, UI/l	568 (424–771)	488 (320–782)	0.559
Free plasma Hb, mg/l	183 (123–282)	130 (107–205)	0.479
Serum iron, μmol/l	16.1 (12.4–19.5)	16.8 (14.9–19.9)	0.849

Record values expressed as frequency or median (IQ 25–75)

Abbreviations: *Hct*: hematocrit, *WB*: white blood cells, *CRP*: Creactive protein, *LDH*: Lactate dehydrogenase, *Hb*: hemoglobin, *HU*: hydroxyurea, *VOC*: vaso-occlusive crisis

*Fisher Exact test

**Fig. 1:**
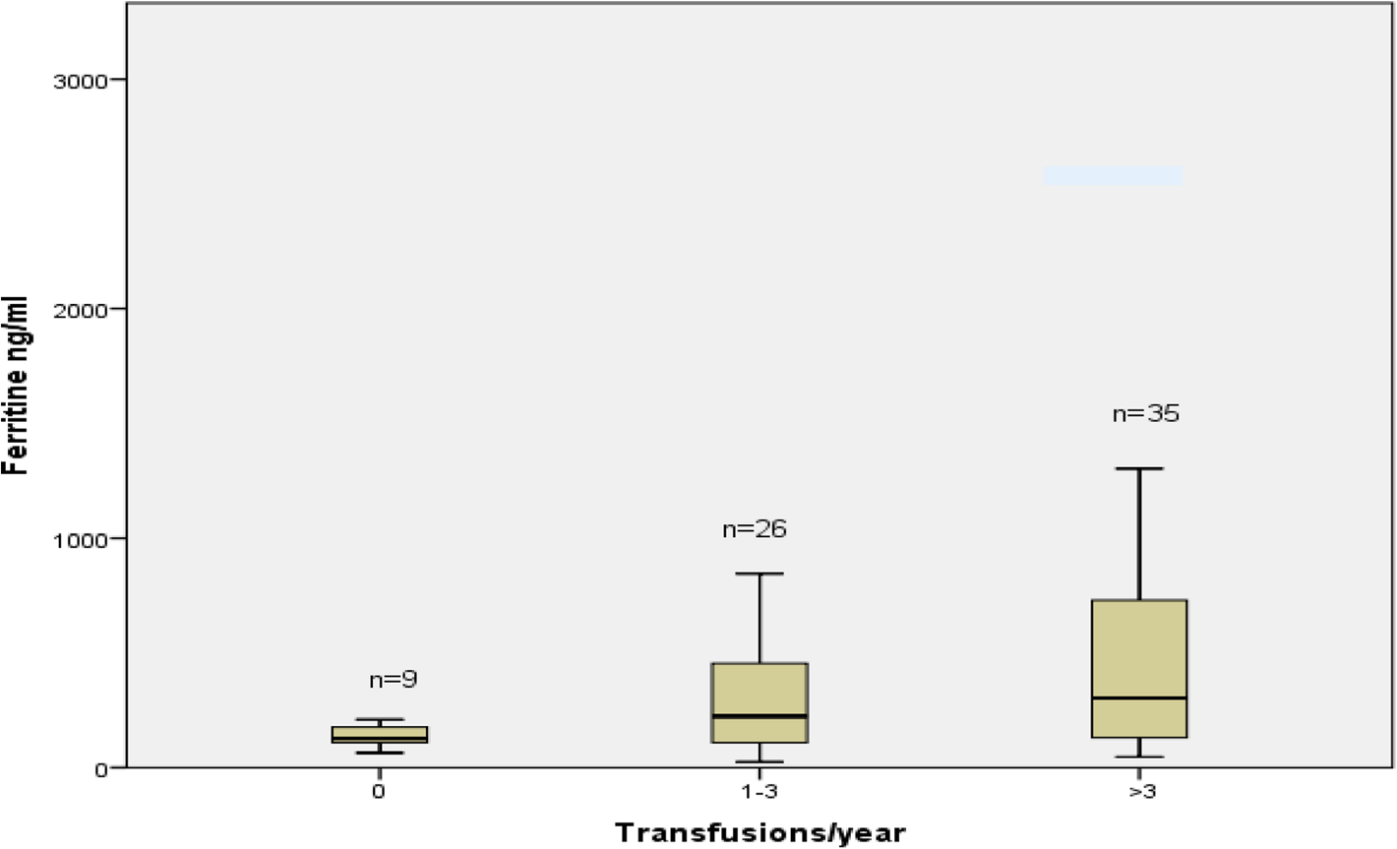
Variation of serum ferritin level as a function of the number of transfusions during the last 12 months

Figure 1 shows that in the whole group, serum ferritin levels increased proportionally with the number of blood transfusions received during the last year; median values of serum ferritin levels were 125 ng / ml in the group 1 (IQR: 99–185 ng/ml), 222 ng / ml in the group 2 (IQR: 102–453 ng/ml), and 301 ng / ml in the group 3(IQR: 123–747 ng/ml) (*p* = 0.010).

Receiving 1–3 transfusions in a year vs. 0 [OR 3.08 (95% CI: 1.06–8.97)] and > 3 transfusions in a year vs. 0 [OR 6.17 (95% CI: 1.81–20.96)] were the only two risk factors of elevated iron stores retained in univariate analysis.

## Discussion

The present study showed that approximately one in five homozygous SCA children had hyperferritinemia level requiring iron chelation. Multiple blood transfusions emerged as the only determinant of elevated iron stores.

In SCA, elevated serum ferritin levels may reflect inflammatory syndrome, hemolysis or transfusion therapy [17]. In the present study, only patients who were in steady state over the last 2 months were selected suggesting a likely reduced impact of inflammation and hemolysis on measured ferritin levels. Furthermore, many studies have reported that increases in serum ferritin levels induced by inflammatory and hemolysis states are usually moderate (< 500 ng/ml) [17, 18]. Higher rates of serum ferritin levels can be seen in alcoholism and some liver diseases not present in our case series [19].

According to several authors, iron overload becomes detectable after 20 blood transfusions [20]. Almost half of our patients and / or their tutors were unable to accurately recall the number of transfusions received since birth, so this assessment was not possible. However, our results indicate that, regardless of the number of blood transfusions received in previous years, children with more than three blood transfusions during the year, experienced elevated iron stores. In a previous Congolese study, Tshilolo et al. showed that the average blood transfusion requirement was 0.4 units per patient-year in Congolese SCA patients [11]. However, our finding need to be strengthen by taking into account of other confounders such as the exact volume of blood received by each patient, the nature of transfused blood (whole blood, red blood cell, single transfusions or erythrapheresis), initial iron status and co-morbidities. Unfortunately, these factors were not available in this post-hoc analysis.

The cumulative and deleterious effects of blood transfusions on iron overload rely upon iron metabolism. Indeed, iron has been reported to lack a physiologic system of elimination exposing it to cumulate in case of increased intake. Thus, during transfusion of red blood cell, nearly 200 mg of iron reach the body; however, every day barely 1 mg of iron is eliminated through the skin and intestinal epithelial: iron is thus eliminated very slowly [4, 20, 21]. The complete saturation of transferrin and ferritin facilitates the binding of iron to other circulating molecules, the basis for the accumulation of reactive iron complexes within parenchymal cells [21]. During the transfer of electrons between iron molecules of different valences (Fe3 + and Fe2 +), extremely reactive free radicals are formed with subsequent oxidative damage of tissues and organs [21, 22].

The frequency of the elevated iron stores ranges from 22 to 41.5% in African studies [11, 14, 23–25]. These differences in the relative proportions of elevated iron stores between these studies presumably arise from differences in the definition of hyperferritinemia, age of the study population, environmental, immunologic and genetic factors that influence iron status in sickle cell population. These situations strongly suggest that more research in African countries would provide valuable insights into the pathogenesis of iron overload as reported in a recent Egyptian cohort [26].

Failure to perform liver biopsy to assess iron overload constitutes a limitation of the present study. In the absence of this invasive technique, imaging techniques such as magnetic resonance and Superconducting Quantum Interference Device (SQUID) have been reported to provide a good correlation between iron tissue concentrations and potential organic damage [27]. However, as a general rule, the serum ferritin concentration assay offers an interesting alternative. Indeed, several studies have shown a linear correlation between serum ferritin levels and total body iron load, based on the results of the assessment of hepatic iron concentration by biopsy analysis [5, 7, 8].

Considering the results of this study, local physicians should be sensitized to avoid iterative transfusions by respecting the indications for blood transfusion in SCA, to perform serum ferritin test, in particular from more than 3 blood transfusions during the year. Indications of iron chelation therapy must be known and systematized to prevent comorbidities associated with iron overload.

## Conclusion

In SCA children, hyperferritinemia requiring iron chelation is most strongly related to blood transfusion. This situation concerned almost one in five children in present study; this shows the magnitude of the problem which is underestimated.

## Abbreviations

CRP: C reactive protein
Hb: Hemoglobin
Hct: Hematocrit
HU: Hydroxyurea
LDH: Lactate dehydrogenase
SCA: Sickle cell anemia
VOC: Vaso-occlusive crisis.
WBC: White blood cell
